# Do Antenatal Parasite Infections Devalue Childhood Vaccination?

**DOI:** 10.1371/journal.pntd.0000442

**Published:** 2009-05-26

**Authors:** A. Desiree LaBeaud, Indu Malhotra, Maria J. King, Christopher L. King, Charles H. King

**Affiliations:** 1 Division of Pediatric Infectious Diseases, University Hospitals of Cleveland, Rainbow Babies and Children's Hospital, Cleveland, Ohio, United States of America; 2 Center for Global Health and Diseases, Case Western Reserve University, Cleveland, Ohio, United States of America; 3 Tufts University, Medford, Massachusetts, United States of America; Retired, Sweden

## Abstract

On a global basis, both potent vaccine efficacy and high vaccine coverage are necessary to control and eliminate vaccine-preventable diseases. Emerging evidence from animal and human studies suggest that neglected tropical diseases (NTDs) significantly impair response to standard childhood immunizations. A review of efficacy and effectiveness studies of vaccination among individuals with chronic parasitic infections was conducted, using PUBMED database searches and analysis of data from the authors' published and unpublished studies. Both animal models and human studies suggest that chronic trematode, nematode, and protozoan infections can result in decreased vaccine efficacy. Among pregnant women, who in developing countries are often infected with multiple parasites, soluble parasite antigens have been shown to cross the placenta and prime or tolerize fetal immune responses. As a result, antenatal infections can have a significant impact on later vaccine responses. Acquired childhood parasitic infections, most commonly malaria, can also affect subsequent immune response to vaccination. Additional data suggest that antiparasite therapy can improve the effectiveness of several human vaccines. Emerging evidence demonstrates that both antenatal and childhood parasitic infections alter levels of protective immune response to routine vaccinations. Successful antiparasite treatment may prevent immunomodulation caused by parasitic antigens during pregnancy and early childhood and may improve vaccine efficacy. Future research should highlight the varied effects that different parasites (alone and in combination) can have on human vaccine-related immunity. To optimize vaccine effectiveness in developing countries, better control of chronic NTDs may prove imperative.

## Introduction

Since the inception of the Expanded Program on Immunization (EPI) in 1974, many global partners, including the World Health Organization, United Nations Children's Fund, and the Gates Foundation, have joined to support mass global immunization projects that have resulted in a significant drop in child mortality worldwide [1]. Programmatic effectiveness has been primarily measured as the operational improvement in vaccine coverage, with the tacit assumption that average individual vaccine response (i.e., average vaccine efficacy) remains the same for all populations [2],[3]. For example, the Global Alliance for Vaccines has improved the percentage of children receiving diphtheria–tetanus–pertussis (DTP) vaccinations from 71% in 1999 to 78% in 2004 [4]. Despite these impressive attempts at mass vaccination coverage, vaccine-preventable diseases still kill an estimated 1 to 2 million African children each year [5]. Whereas efficacy is the measure of the impact of treatment in an ideal (study) environment, effectiveness is the measure of impact in “real-world” settings [6]. These preventable deaths contribute to the high infant and childhood mortality rates experienced by these countries and, by definition, highlight the lapse in vaccine effectiveness in resource-poor areas.

**Figure 1:**
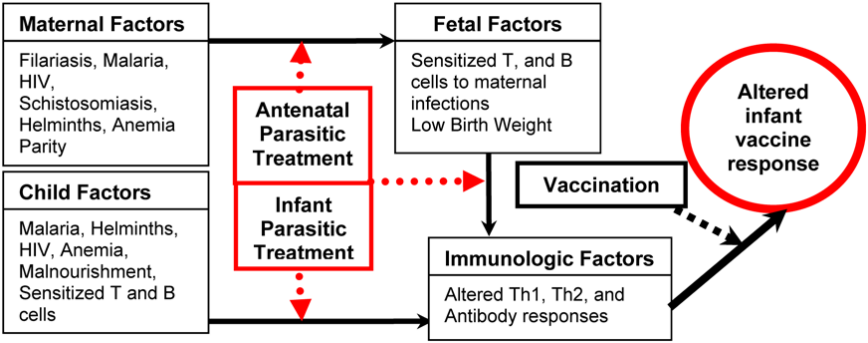
Theoretical mechanisms of reduced vaccine response in infants.

Vaccines are among the most cost-effective health interventions available for the prevention of life-threatening and disabling infectious diseases. Even so, the overall effectiveness of vaccine strategies requires both adequate coverage among vulnerable populations and induction of a satisfactory protective immune response in each susceptible individual. Although extensive resources are now being committed to improve global childhood vaccination coverage, in developing nations the response to standard vaccination often remains suboptimal [2], [7]–[12]. The reasons for this poor vaccination response are undoubtedly complex, yet there are several causes that are likely to be amenable to intervention or preventive treatment (Figure 1). In particular, emerging clinical evidence suggests that chronic antenatal parasitic infection can significantly alter infant immune responses to standard childhood vaccinations [13]–[21]. More limited evidence also suggests that parasitic infections in the first few years of life can also impact immunity and response to vaccines [22]. In this review, our premise is that vaccine effectiveness will not be optimal among children of developing countries until there is adequate treatment and prevention of antenatal and early childhood parasitic infections.

The reduced effectiveness of vaccination programs in developing communities often has been blamed on cold-chain lapses and a lack of support infrastructure [23]–[27]. However, as detailed later in this review, chronic infections with neglected tropical diseases (NTDs) also appear to play a significant role in poor vaccine efficacy. Of special interest, maternal parasitic infections affect the unborn infant and appear to act as important immune response modifiers, although the mechanisms of the parasite-induced immune effects are not yet fully understood. Current evidence suggests that maternal parasitic infections such as schistosomiasis [28], filariasis [28]–[30], other helminths [31], and malaria [32] during the period of gestation can suppress an infant's later immune responses to standard childhood vaccinations.

## Population-Based Evidence of Reduced Vaccine Effectiveness

Several vaccination studies have shown that children from areas of sub-Saharan Africa are less responsive to standard childhood vaccines than children from developed countries. These include vaccine trials of the antituberculosis vaccine BCG (Bacillus Calmette-Guerin) [7],[8],[33] and of typhoid fever [9], measles [10],[23], and polio vaccines [11],[12]. For example, after receiving three oral polio vaccines, vaccinated children from industrialized nations have 97%, 100%, and 100% seroconversion rates to polio virus types 1, 2, and 3, whereas vaccinated children from developing nations show only 73%, 90%, and 70% protection, respectively [11].

What are the implications of this phenomenon? Recent outbreaks of polio in Africa show us that our “control” of vaccine-preventable diseases is tenuous at best. A short-term failure in vaccine coverage resulted in the rapid and serious resurgence of a vaccine-preventable disease [34]. In recent Nigerian polio outbreaks, the rapid spread to nearby African countries was in part related to low herd immunity caused by decreased vaccine coverage (10 countries) [35]. Of special importance, however, polio also reemerged in well-vaccinated African countries such as Ghana, Botswana, and, now more recently, Kenya, where the vaccine coverage rate was high (>90%) [35],[36]. Vaccine programs in these countries were ineffective in preventing these outbreaks, despite high vaccination coverage, suggesting suboptimal vaccine efficacy among at-risk individuals within well-vaccinated local populations.

## Potential Causes of Reduced Efficacy in Developing Countries

Failure to respond appropriately to vaccination is most often associated with a suite of poverty-related conditions, including both malnutrition and chronic infection. Clinical features of poverty include protein-calorie and micronutrient undernutrition and recurring exposure to parasites (protozoa and helminths) that independently contribute to chronic anemia and poor physical growth and development [37]. Chronic parasitic infections also have a substantial impact on cognitive and intellectual development and education [38]–[40]. Fortunately, many of these deleterious effects can be reversed with antiparasite treatment [41]–[44]. However, reinfection remains common in this setting, and the global burden of parasitic infections remains unacceptably high [45].

## Effects of Parasitic Infection on Host Immunity

The deleterious effects of polyparasitism on host immunity may help to explain the poor response to childhood vaccination that is seen in the developing world. Both animal and human studies indicate that parasitic infections can impair long-term responses to vaccination. In addition, multiple concurrent infections are likely to have additive or synergistic effects on immune responses. Studies in both human and animal models have shown that trematode, nematode, and *Plasmodium* infections lead to decreased vaccine efficacy and an inability to ward off new infection [19], [46]–[48].

### Trematode Infections

Elias et al. [15] compared the efficacy of BCG vaccine in mice with and without *Schistosoma mansoni* infection and determined that BCG-vaccinated, schistosome-infected mice have significantly less vaccine-induced protection against virulent tuberculosis challenge than those without schistosome infection. In studies of humans with trematode infection, a decreased response to tetanus toxoid vaccination has been demonstrated in the presence of schistosomiasis, associated with significantly altered Th1- and Th2-type immune responses to tetanus toxoid in vitro [18].

### Nematode Infections

Two studies performed in mice show that nematode infection impairs response to malaria vaccination and further illustrate that cure of parasitic infection before immunization, and not after, increases host vaccine response [18],[46],[47]. Additionally, in humans, deworming with albendazole has been shown to improve the effectiveness of vaccine response to BCG [16] and to oral cholera vaccine [13],[14].

### Protozoan Infections

Chronic protozoan infections also have been proven to have harmful effects on vaccination response, with decreased levels of response to tetanus, *Haemophilus influenzae* type b [5], and typhoid vaccines noted in the presence of concurrent malaria infection [17],[20],[21]. Whether or not treatment of malaria can improve vaccine response is controversial and appears to depend on which vaccines are tested. No effect was observed on DTP and measles vaccination [49],[50], but malaria treatment has been found to be associated with improved immunization response to meningococcal vaccines [51],[52].

## Evidence on the Impact of Antenatal Parasite Infection on Later Childhood Vaccine Responses

**Figure 2:**
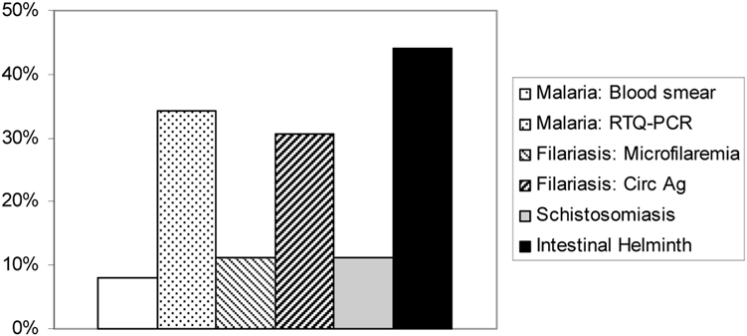
Typical profile of parasitic infection prevalence among pregnant women attending an antenatal clinic in coastal Kenya. Dual infection was detected in 26% of women; three or more infections were detected in 11% of women.

**Figure 3:**
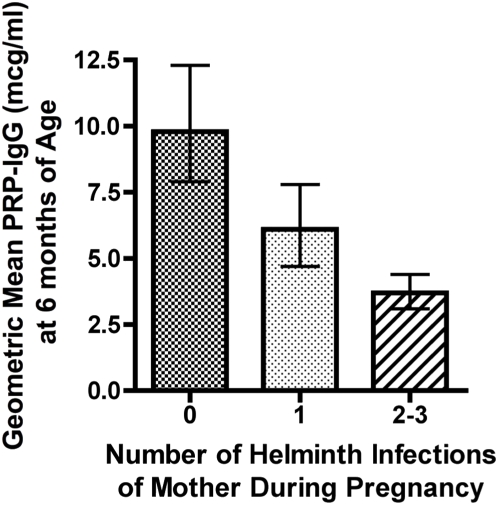
Effect of maternal helminth infection on an infant's acquisition of protective antibodies (polyribosylribitol phosphate-specific IgG) following *Haemophilus influenzae* type b vaccination at 6, 10, and 14 wk of age. Values represent geometric means (±95% confidence interval) for infants subsequently tested at 6 months of age. *p<0.001 for observed differences between the offspring of uninfected versus multiply parasitized women.

“Imprinting” of the immune system during fetal development also may play a significant role in reduced vaccine effectiveness in parasite-endemic areas. In developing countries, women of child-bearing age are frequently infected with one or more parasites, as seen in our cohorts in coastal Kenya and others [53] (Figure 2). If left untreated, their chronic infections will persist throughout the period of pregnancy. As a consequence of persistent infection, soluble parasite-derived products cross the placenta, potentially priming or tolerizing the fetal immune system's response to these parasite-specific antigens and to unrelated antigens [31]. Many helminth-mediated immunoregulatory effects drive the balance of human host immunity towards Th2-type reactivity, yet natural or vaccine-mediated immunity requires mainly Th1-type responses to provide vaccine-mediated protection [30],[54]. On the basis of emerging evidence from longitudinal maternal–infant cohort studies, exposure to parasites in utero appears to induce an immunomodulatory phenotype that persists into infancy and later childhood, significantly affecting protective responses to antigens included in standard childhood vaccines (Figure 3). The treatment of helminthic infections during pregnancy has been shown to have many beneficial effects, including the reduction of HIV mother-to-child transmission (MTCT), low birth weight incidence, and infant mortality [55]. Data suggest many other detrimental effects of chronic maternal parasitic infection on infant outcomes [55]–[64]. Whether or not parasitic treatment of pregnant women can impact infant immunization efficacy has not been adequately studied, although evidence suggests that treatment of parasitic infections in pregnant women has significant immunity-modifying effects in their infants [28],[65].

## Evidence on the Impact of Early Childhood Parasitic Infection on Vaccination Responses

Successful treatment of pregnant mothers for parasitic infections may prevent sensitization to parasitic antigens during pregnancy and may improve vaccination outcomes of the unborn child by diminishing parasite-driven fetal priming. Later primary exposure to parasitic infections during the first years of life also can affect subsequent immune response to vaccination. Although malaria is the most common parasitic infection in young children, schistosomiasis, filariasis, and intestinal helminthic infections also have been shown to occur in infants [30],[66],[67]. Polyparasitism is known to affect immune function and vaccination response [15],[18],[19],[46],[47]. The evidence that early childhood parasitic treatment yields improved response to vaccination is sparse and deserves further study [16],[51],[52].

## Proposed Mechanisms

Studies have clearly established the presence of T and B cell responses by the human fetus to maternal parasitic infections [32], [68]–[70]. Animal models suggest that exposure to parasites in utero may be either beneficial (by accelerating the development of protective antibodies and cellular immune responses) or detrimental (by impairing the acquisition of a protective immune response and inducing immune tolerance) [71]–[74].

Immune tolerance may be due to clonal deletion of specific cell subsets, which leads to loss of antigen-reactive cells in utero and subsequent lack of recall response during antigen exposure later in infancy. Another possible tolerance mechanism is clonal anergy, which results from a lack of adequate costimulation by antigen-presenting cells to CD4+ cells. Finally, tolerance may be due to the de novo generation of populations of immunoregulatory cells. Prenatal exposure to malaria, schistosomiasis, and filariasis may lead to the acquisition of a subset of immunoregulatory CD4+ T cells. A distinct population of CD4+ regulatory-1 cells (Tr1) has been shown to be able to arise from CD34+ cells in the neonate [75] in response to IL-10 and IFN-α [76]. Specifically, IL-10 has been proposed to be responsible for modulating subsequent immune responses during subsequent exposure to new, unrelated antigens. Tr1 cells are antigen-specific CD4+ cells that are distinct from Th1 and Th2 cells in that they produce significant amounts of IL-10, variable IFN-γ, TGF-β, and IL-5, but little or no IL-2 and no IL-4. The CD4+CD25+ T cells represent another population of regulatory T cells whose development is partially mediated by IL-10 and TGF-β [77]. As with chronic helminth infections, exposure to maternal malaria leads to the generation of significant levels of malaria blood stage antigen-driven IL-10 in cord blood and infant lymphocytes. Presumably, this immunomodulation may benefit the human host by limiting inflammation caused by chronic parasite infections in early childhood and later adolescence. However, the concomitant downside is a reduced ability to respond to acute bacterial and viral infections and to vaccination.

In humans, “tolerance” (manifested by the generation of immunoregulatory populations of lymphocytes by prenatal antigenic exposure) is likely to contribute to the long-term persistence of many intravascular parasitic infections [30], [54], [78]–[80]. In our studies, tolerance is defined as an altered or suppressed immune response to parasite antigens during infancy or childhood in the progeny of women infected during pregnancy. Maternal infection with lymphatic filariasis, schistosomiasis, and the protozoan parasites *Trypanosoma cruzi* and *T. gondii* have been shown to enhance the offspring's susceptibility to subsequent infection, and this phenomenon is associated with impaired or altered fetal immune response to parasite antigens. Children born to filarial-infected mothers, for example, have depressed cytokine responses in T cells and lymphocyte proliferation by peripheral blood mononuclear cells to filarial antigens compared to offspring from uninfected mothers. They are also significantly more likely to acquire filarial infection in the first 5 years of life and continue to show altered responses to parasite antigens years after birth [29].

Not all in utero exposure to maternal helminth infections results in tolerance. Instead, for some newborns, prenatal parasite exposure results in a constant state of antiparasite immune activation that is characterized by a Th2-dominant cytokine profile, high IgE levels, and eosinophilia. Such an immune profile also may have an adverse impact on the efficacy of vaccines by limiting Th1 pathways of immune response to vaccination. By altering the immunologic balance between Th1 and Th2 pathways, chronic parasitic infections appear to alter the immunologic milieu and would also likely impair or suppress the “normal” responses to vaccines that have been described in parasite-free, developed countries.

## Other Implications of Parasite Infection for Infectious Disease Transmission

Polyparasitism's effects on immunity are believed to be partly responsible for the increasing virulence of the world's most lethal infections: HIV [81],[82], malaria [83],[84], and tuberculosis [85],[86]. In addition, helminth infection is associated with an increased risk for MTCT of HIV [87]. Some studies of HIV–parasite coinfection suggest that antihelminthic therapy may reduce CD4 depletion and progression of viral load in HIV-infected patients [88]. Parasite effects on immunity need to be highlighted as new vaccines against HIV, malaria, and tuberculosis are moving forward in clinical trials, because optimal vaccine efficacy may require effective antiparasite therapy to control for the immune system impact of these chronic pathogens [38], [89]–[92].

## Suggestions for Current Antenatal and Infant Care and for Future Research

A growing body of evidence now demonstrates that antenatal and childhood parasitic infections both deleteriously alter responses to routine vaccination. To truly optimize vaccination campaign effectiveness and vaccine efficacy in all areas of the world, parallel efforts are needed to control endemic parasitic infections. More data are needed to identify the multifactorial causes of impaired vaccine efficacy, including research specifically addressing the impact of preventive antiparasite therapy on maternal and infant health outcomes.

The immune response to parasites can be beneficial or detrimental. Immune modulation makes it difficult to predict the impact of intermittent parasite treatment. Perhaps a repeating cycle of infection/treatment/reinfection may be worse for vaccine response than low level chronic infection. This area is worthy of further study and must determine the appropriate treatment intervals for these infections. Also, more data are needed to objectively define the optimal timing between deworming and vaccination. Ideally, pregnant women should be free of infection throughout all three trimesters of pregnancy, but perhaps reinfection in the last trimester is particularly detrimental with respect to fetal priming. Ultimately, a carefully designed double-blind, placebo-controlled trial in infected pregnant women would provide the critical evidence to support or refute the need for antenatal parasitic treatment.

If antenatal and early child deworming are to be implemented as standard interventions, then the associated changes in vaccine efficacy and effectiveness should be concurrently documented in well-designed, prospective studies. Animal models suggest that the loss of cross-protective or immunomodulatory effects of polyparasitism may mean that some parasitic diseases may become clinically worse before they are fully eradicated. So far, the available evidence indicates that deworming has clear benefits in terms of birth outcomes [62],[93]. If deworming is also found to amplify vaccination effects, then it is clearly time to intensively test and implement antiworm strategies and their “indirect vaccination” effect on at-risk populations. Ultimately, because of the real-world roadblock that parasite infections present to vaccine effectiveness, mass vaccination campaigns likely will not reach their full potential unless vaccines and antiparasite strategies are implemented together.
